# An Explainable Intelligent Fault Diagnosis for Rotating Machinery via Multi-Source Information Fusion Under Noisy Environments and Small Sample Conditions

**DOI:** 10.3390/s26051713

**Published:** 2026-03-08

**Authors:** Gaolei Mao, Jinhua Wang, Yali Sun

**Affiliations:** 1 School of Automation and Electrical Engineering, Lanzhou University of Technology, Lanzhou 730050, China; 232085406039@lut.edu.cn (G.M.); 242085406083@lut.edu.cn (Y.S.); 2 School of Microelectronics Industry-Education Integration, Lanzhou University of Technology, Lanzhou 730050, China

**Keywords:** fault diagnosis, rotating machinery, small sample conditions, multi-sensor data intelligent fusion, multi-scale Swin Transformer

## Abstract

In modern industrial systems, the fault diagnosis of rotating machinery is crucial for ensuring safe equipment operation. However, practical fault data are often contaminated by noise, and the scarcity of samples across fault conditions makes effective feature extraction challenging. Moreover, single-sensor measurements provide limited and incomplete information, further degrading the accuracy and reliability of diagnostic models. To address these challenges, this paper proposes an explainable intelligent fault diagnosis for rotating machinery via multi-source information fusion under noisy environments and small sample conditions. Firstly, a multi-sensor data intelligent fusion module (MSDIFM) is developed. It converts multi-sensor vibration signals into time–frequency maps via continuous wavelet transform (CWT). Pixel-level cross-channel fusion is then performed using a variance-driven dynamic weighting strategy to generate a unified fusion map, adaptively highlighting high information channels. Secondly, a multi-dimensional adaptive asymmetric soft-threshold residual shrinkage block (MASRSB) is proposed to implement differentiated and dynamic threshold control for positive and negative features, enhancing representation and discrimination capabilities. Thirdly, the multi-scale Swin Transformer (MSSwin-T) is designed. This module significantly enhances the model’s feature extraction capability by expanding multi-level receptive fields, strengthening key channel representations, and reinforcing cross-window feature interactions. Finally, to validate the effectiveness of the proposed method, experiments are conducted on both the Case Western Reserve University (CWRU) dataset and the self-created PT890 dataset. Results demonstrate that the proposed method exhibits outstanding diagnostic performance and robustness under noisy conditions and with small sample sizes.

## 1. Introduction

With the advancement of the information era and intelligent manufacturing, rotating machinery has found widespread adoption in critical sectors, including transportation, energy systems, and process industries. As core supporting and transmission components, the health of rolling bearings directly determines the operational safety and efficiency of the machine [1,2,3]. Fault can cause unplanned shutdowns, equipment damage, casualties, and substantial economic losses [4,5]. Therefore, rapid and reliable bearing fault diagnosis is of significant engineering value and practical urgency [6].

In equipment condition monitoring and fault diagnosis, traditional approaches emphasize signal processing-based feature engineering. For raw vibration or current signals, common processing methods include the following: the Fourier transform is primarily used for frequency domain analysis of signals. By converting time domain signals into frequency domain signals, it identifies periodic components and characteristic frequencies within the signal [7]. Adaptive decomposition of non-stationary signals using empirical mode decomposition (EMD) enables the extraction of local metrics such as instantaneous energy and instantaneous frequency [8]. The wavelet transform enables multi-scale analysis in the time–frequency domain, providing detailed local characterization of signals and highlighting transient impacts and localized damage features [9]. Building on this foundation, researchers typically identify features that are highly correlated with faults and combine them with classifiers to improve diagnostic accuracy. Tang et al. [10] developed a supervised K-nearest neighbor (KNN)-based fault diagnosis approach and achieved a diagnostic accuracy of 98.9% on a wind turbine. Li et al. [11] jointly estimated the fault cycle and filter length using the envelope harmonic-to-noise ratio (EHNR) and spectral entropy as criteria. Wang et al. [12] proposed a time–frequency analysis-based fault diagnosis method validated on two datasets, showing strong robustness to noise. Although the aforementioned methods can accurately identify fault types, they rely heavily on handcrafted features and empirically tuned parameters, leading to performance drift and limited generalization under varying operating conditions. In recent years, deep learning has gained prominence, replacing handcrafted features with end-to-end representation learning. It directly extracts features adaptively from raw signals or time0frequency plots, demonstrating promising applications in the field of fault diagnosis.

As a representative deep learning architecture, convolutional neural networks (CNNs) [13] possess adaptive feature learning capabilities and have achieved notable success in fault diagnosis. Wang et al. [14] proposed an adaptive denoising convolutional neural network (ADCNN) that integrates an adaptive denoising unit. This approach suppresses noise while preserving salient fault features, thereby eliminating the need for manual configuration of denoising functions and improving fault diagnosis efficiency. Wang et al. [15] transform multivariate process data into a curve-like representation using the Andrews plot and utilize a CNN to achieve intelligent process fault diagnosis. This approach effectively reduces the reliance on manual feature design and improves the diagnostic performance. Zhang et al. [16] employed adaptive optimization of adaptive multivariate variational mode decomposition (AMVMD) parameters based on the minimum modal overlap criterion. They first decomposed the vibration signal for noise reduction, then utilized a multi-scale convolutional neural network for feature extraction and classification. Feng et al. [17] integrate time–frequency attention, LMMD subdomain alignment, and contrastive local alignment to propose an unsupervised subdomain contrastive adaptation method, which improves cross-domain diagnostic capability under unlabeled target-domain conditions. Liang et al. [18] introduced a multi-scale dynamic adaptive residual network with attention-based multi-scale convolutions that adapt weights online to strengthen feature extraction. However, as network depth increases, vanishing gradients can still arise, limiting training stability and overall performance in CNN-based intelligent diagnostics. Residual neural networks (ResNets) [19] introduce identity shortcut connections that bypass intermediate layers, thereby preserving gradient flow during backpropagation. This design substantially mitigates vanishing gradients in deep CNNs and has been widely adopted for rolling bearing fault diagnosis because of its stable performance. Wu et al. [20] proposed a time–frequency residual convolutional neural network (TFRCNN) with dual branches operating in the time and frequency domains to extract features independently, thereby improving representational completeness. They introduced tailored residual units to mitigate degradation in deep networks and employed global average pooling to promote model sparsity and improve generalization. Lad et al. [21] developed a deep neural network for end-to-end characterization of motor fault data, uncovering fine-grained features that are difficult to capture with traditional methods. They then employed multiple support vector machines (SVMs) for decision-level fusion, thereby enhancing discriminative power and enabling robust fault diagnosis. Tong et al. [22] proposed the following two modules: pseudo soft thresholding and adaptive slope to suppress signal distortion. Building on these modules, they constructed an improved residual shrinkage unit that adaptively sets thresholds and slopes, demonstrating greater robustness in high noise scenarios. Zhuang et al. [23] designed a fault diagnosis framework based on an improved multi-scale residual network, enhancing the feature retention capability of signals from different modalities and achieving improvements in both noise suppression and recognition performance. Zhu et al. [24] combined four one-dimensional sensor signals into a single signal and employed a deep Q-network for feature learning, demonstrating that this approach maintains good diagnostic performance even with small sample sizes. Shao et al. [25] employed a dual threshold attention-guided framework to enable a generative adversarial network (GAN) to prioritize focusing on globally relevant thermal information during training, thereby enhancing the quality of generated images. Chen et al. [26] constructed a generator framework combining CNN and Transformer architectures, employing micro augmentation and synthetic minority class oversampling strategies to simultaneously expand the dataset and enhance its diversity. In summary, although multi-sensor fault diagnosis methods have achieved certain results, challenges remain in achieving efficient feature extraction and accurate fault diagnosis under noisy conditions and in scenarios with limited data samples.

Therefore, to address the degraded diagnostic accuracy and reliability under noisy environments and small sample conditions, this paper proposes an intelligent rolling bearing fault diagnosis method for small sample conditions based on explainable multi-source information fusion. The main contributions of this paper are as follows:

(1) Compared with commonly used multi-sensor fusion strategies, we propose an MSDIFM. It transforms each sensor signal into a CWT time–frequency map and performs variance-driven channel weighting with pixel-level fusion to generate a unified fused map. This design clearly emphasizes the information-rich sensors, while suppressing the channels with less information or those affected by noise. Moreover, the learned weights provide a clear basis for explaining the functions of the sensors.
(2) Compared with conventional residual shrinkage blocks that typically employ symmetric thresholding and limited shrinkage behaviors, we construct a MASRSB. It integrates a multi-scale feature extraction module, a dual-attention asymmetric threshold block, an adaptive threshold slope module, and a polarity-aware adaptive asymmetric soft-threshold function. By separating the positive and negative responses and performing dynamic threshold and slope adjustments, MASRSB is able to suppress noise while better preserving the weak but discriminative fault components.
(3) Compared with directly applying vanilla Swin-Transformer blocks to time–frequency images, we design a tailored multi-scale Swin Transformer (MSSwin-T) for fault diagnosis. Specifically, a multi-scale feed-forward network (MS-FN) combines deep convolutions with dual attention to enhance channel aggregation and expand receptive fields at multiple scales, while a shifted-window multi-head self-attention (SWHSA) adopts an improved window shifting strategy to strengthen cross-window feature interaction. These enhancements jointly improve feature extraction and generalization under noisy and small-sample conditions.

The remainder of this paper is organized as follows. Section 2 reviews the relevant theories. Section 3 presents the proposed methods. Section 4 provides experimental validation and analysis. Section 5 concludes the paper.

## 2. Relevant Theories

### 2.1. Continuous Wavelet Transform (CWT)

Rolling bearing vibration is a prototypical nonstationary signal that exhibits complex time–frequency characteristics. Among time–frequency analysis techniques, the CWT [27] efficiently maps one-dimensional time series signals to two-dimensional time–frequency maps. This method offers robust multi-scale feature extraction capabilities, accurately characterizing feature distributions across temporal and frequency scales, and exhibits strong localization, making it particularly suitable for analyzing nonstationary signals. Leveraging these advantages, we employ the CWT to convert one-dimensional vibration signals into two-dimensional time–frequency maps, thereby enabling the precise extraction of rolling bearing fault characteristics.

### 2.2. Transformer

**Figure 1:**
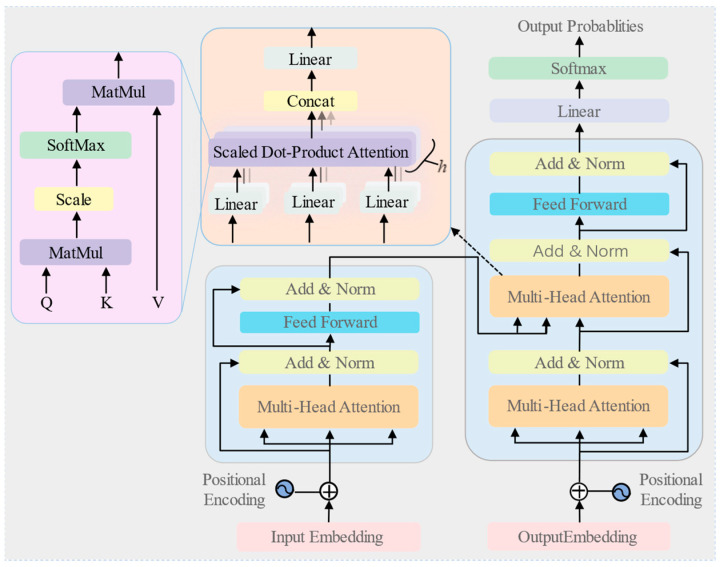
Schematic of the Transformer architecture.

The Transformer was introduced by Vaswani in 2017. It adopts an encoder–decoder architecture for sequence-to-sequence tasks. The encoder maps the input sequence to a latent representation, while the decoder progressively generates the target sequence by conditioning on the encoded representation and the previously generated tokens. Figure 1 illustrates the overall architecture. Within the Transformer, the encoder and decoder share many structural components. Both incorporate multi-head self-attention (MHSA) and feed-forward networks, while also utilizing layer normalization and residual connections.

## 3. Proposed Method

### 3.1. Multi-Sensor Data Intelligent Fusion Module (MSDIFM)

**Figure 2:**
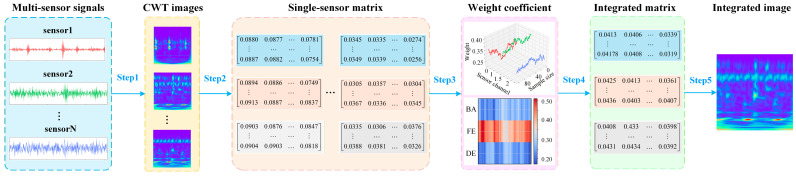
Architecture of the MSDIFM.

During vibration data acquisition for rotating machinery, multiple sensors are typically deployed for synchronous data collection. However, many existing diagnosis methods still rely on a single sensor or multi-channel direct superposition, which may dilute fault-sensitive channels and introduce redundancy under noise. To obtain richer and more reliable representations, we propose an MSDIFM, as shown in Figure 2. MSDIFM assigns data-driven reliability weights to different sensors and performs pixel-level fusion on CWT time–frequency maps, thereby constructing an explicit and explainable multi-source fusion representation. The module comprises the following key steps.

First, apply a CWT to each sensor’s data, converting its one-dimensional signal into a two-dimensional time–frequency image. Then, compute the absolute value of each sensor’s CWT image and perform normalization, mapping pixel values to the range [0, 1] to obtain a single sensor pixel matrix. Next, the variance of each sensor’s data segment is computed as a dynamic weighting metric, yielding normalized weight coefficients for each channel. Subsequently, these channel weights are applied to perform pixel-level fusion on the pixel intensity matrices of each sensor within the time–frequency map, constructing a multi-source information fusion matrix. Finally, the fused pixel matrix is mapped to generate a unified multi-sensor fusion time–frequency image.

Existing fusion strategies such as channel stacking or decision-level fusion often implicitly assume comparable reliability across sensors. In contrast, MSDIFM assigns a variance-driven weight to each sensor segment and conducts pixel-wise fusion on the corresponding CWT maps, thereby explicitly amplifying fault-sensitive channels while attenuating less informative ones. In addition, the learned weights are visualizable, offering direct and interpretable evidence of each sensor’s contribution.

### 3.2. Multi-Dimensional Adaptive Asymmetric Soft-Threshold Residual Shrinkage Block (MASRSB)

**Figure 3:**
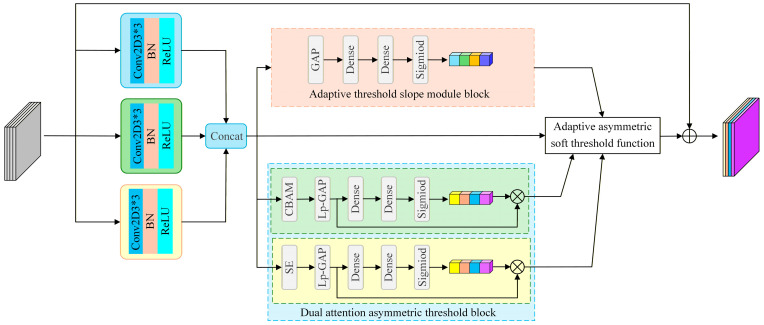
Architecture of the MASRSB.

Traditional soft threshold residual shrinkage blocks apply a soft thresholding function that zeros coefficients within the threshold band and shrinks those outside the band toward zero. Although this operation encourages sparsity and improves computational efficiency, it can also remove subtle yet important information, particularly in tasks that require preserving low-amplitude features. Additionally, the distributions of positive and negative features in fault signals may differ. In conventional designs, using a single global threshold can hinder learning of asymmetric and fine-grained features. Therefore, we propose a MASRSB, whose module architecture is shown in Figure 3. The block comprises an adaptive asymmetric soft-threshold function, a multi-scale feature extraction module, a dual attention asymmetric threshold block, and an adaptive threshold slope module.

#### 3.2.1. Adaptive Asymmetric Soft-Threshold Function (ASTF)

Traditional residual shrinkage networks employ soft thresholding functions that zero coefficients within a threshold band and shrink those outside toward zero. The conventional soft thresholding function is given by Equation (1):

(1) $$f\left(x\right)=\left\{\begin{array}{cc} \mathrm{sgn}\left(x\right)\cdot \left(\left|x\right|-\omega \right) & \left|x\right|\geq \omega \\ 0 & \left|x\right|<\omega \end{array}\right.$$

where $\mathrm{sgn}\left(\cdot \right)$ denotes the sign function.

Although the soft thresholding function in Equation (1) yields a continuous output, several issues remain. First, conventional soft thresholding employs a single global threshold, making it difficult to accommodate distributional differences between positive and negative signal intervals. When noise levels or feature amplitudes differ markedly between positive and negative signals, a fixed threshold can produce imbalanced denoising across signs. Second, for x≥ω, conventional functions impose a constant bias between the input and output, which can cause loss or distortion of high amplitude features. This is particularly detrimental for bearing fault diagnosis, where preserving impact-related high-amplitude features is essential. Finally, these functions set the output to zero when x<ω, thereby discarding all sub-threshold values. However, in real-world settings, values removed by this filtering may still carry important information. Therefore, to address these issues, we propose an adaptive asymmetric soft-thresholding function, whose analytical form is

(2) $$f(x)=\left\{\begin{array}{cc} \mathrm{sgn}\left(x\right)\cdot \left(1-e^{-\beta (|x|-\omega_{1})}\right) & x\geq \omega_{1}\\ \beta \cdot x & \omega_{2}<x<\omega_{1}\\ \mathrm{sgn}\left(x\right)\cdot \left(1-e^{-\beta (|x-\omega_{2}|)}\right) & x\leq \omega_{2} \end{array}\right.$$

where $w_{1}$ denotes the positive threshold, $\omega_{2}$ denotes the negative threshold, and β denotes a learnable parameter that adjusts the compression intensity in the intermediate interval and modulates the nonlinear compression level outside the threshold regions.

The proposed adaptive asymmetric soft-threshold function combines linear and nonlinear compression while preserving continuity for both positive and negative inputs. In the mid-amplitude region, a learnable parameter β controls partial feature retention, whereas the high-amplitude region applies exponential decay to suppress extreme values. This design preserves critical diagnostic information while suppressing noise and reducing redundancy.

#### 3.2.2. Multi-Scale Feature Extraction Module

To extract discriminative features with greater precision, we propose a multi-scale feature extraction module. The module employs a hierarchical, parallel convolutional architecture that enables adaptive fusion of multi-scale features, addressing the limitations of traditional methods, namely, single-scale processing and inefficient feature integration.

To further expand the receptive field and improve feature extraction efficiency, we design three-scale network architecture. This architecture performs multi-scale feature extraction by integrating 3 × 3, 5 × 5, and 7 × 7 convolutional kernels, thereby broadening the effective receptive field of the feature maps. In general, 3 × 3 kernels efficiently capture fine details and local structure, whereas larger 5 × 5 and 7 × 7 kernels are better suited to modeling broader context and global structure. Moreover, 3 × 3 kernels are more computationally efficient, whereas 5 × 5 and 7 × 7 kernels provide more comprehensive contextual coverage. By combining these multi-scale kernels in parallel, the model attains stronger feature extraction performance while maintaining computational efficiency.

#### 3.2.3. Dual-Attention Asymmetric Threshold Block (DA-ATB)

To enhance the representational capacity and differential sensitivity of positive and negative feature channels during the shrinkage stage, we introduce a differentiated attention mechanism: Squeeze and Excitation (SE) channel attention is applied to the positive branch to amplify salient channel representations, whereas spatial attention is applied to the negative branch to increase sensitivity to weak or locally anomalous features.

Assume the input feature is x, where the positive polarity feature is $x_{+}$ and the negative polarity feature is $x_{-}$. Perform absolute value operations on the positive polarity features, then feed them into the channel attention module to obtain channel-weighted features. Then, the soft vector $k_{1}$ applied to the positive channel undergoes a GAP operation. Subsequently, through two fully connected layers and sigmoid activation functions, the positive channel’s adjustable factor $h_{1}$ is obtained. Finally, by multiplying the positive soft vector by the positive channel’s adjustable factor, the positive channel’s threshold $w_{1}$ is derived. Its mathematical expression is

(3) $$k_{1}=GPA\left(SE\left(x_{+}\right)\right)$$

(4) $$h_{1}=F_{sig}\left(F_{fc}\left(F_{fc}\left(k_{1}\right)\right)\right)$$

(5) $$\omega_{1}=k_{1}\cdot h_{1}$$

where $GPA\left(\cdot \right)$ denotes GAP, $F_{fc}\left(\cdot \right)$ denotes a fully connected layer, and $F_{sig}\left(\cdot \right)$ denotes the sigmoid function.

The processing procedure for the negative polarity feature $x_{-}$ is identical to that for the positive polarity feature $x_{+}$. The mathematical expression for this process is

(6) $$k_{2}=GPA\left(SA\left(x_{-}\right)\right)$$

(7) $$h_{2}=F_{sig}\left(F_{fc}\left(F_{fc}\left(k_{2}\right)\right)\right)$$

(8) $$\omega_{2}=k_{2}\cdot h_{2}$$

where $k_{2}$ denotes the negative soft vector, $h_{2}$ represents the negative dynamic adjustable factor, and $\omega_{2}$ indicates the negative threshold.

By applying differentiated attention to positive and negative channels and processing the two polarities separately, the model more effectively captures polarity-specific heterogeneity, thereby improving network robustness in noisy conditions.

#### 3.2.4. Adaptive Threshold Slope Module (ATSM)

Assuming an input feature tensor x, the module first applies GAP to extract channel-wise statistics. The pooled vector then passes through two fully connected layers, followed by a sigmoid activation, to produce the adaptive adjustment factor β for the positive and negative thresholds. Formally, the process can be expressed as

(9) $$\beta =F_{sig}\left(F_{fc}\left(F_{fc}\left(GPA\left(x\right)\right)\right)\right)$$

Difference to conventional residual shrinkage blocks: existing shrinkage units usually adopt symmetric thresholds and a single shrinkage profile, which can lead to imbalanced denoising and over-suppression of weak fault cues. MASRSB explicitly decouples positive and negative responses and introduces polarity-aware adaptive asymmetric thresholds with learnable slope modulation, enabling differentiated suppression and preservation behaviors across feature polarities. Combined with dual-attention threshold estimation and multi-scale extraction, MASRSB provides more flexible noise suppression while retaining discriminative weak components that are critical for small-sample diagnosis.

### 3.3. Multi-Scale Swin-Transformer (MSSwin-T)

Instead of directly adopting vanilla Swin Transformer blocks for time–frequency fault diagnosis, we design a tailored multi-scale Swin Transformer (MSSwin-T) to address two bottlenecks: insufficient channel aggregation in the feed-forward unit and limited cross-window interaction for non-stationary time–frequency patterns under noise and varying operating conditions. MSSwin-T enhances the Swin backbone with two components: a multi-scale self-attention enhanced feed-forward network (MS-FN) and an improved shifted-window self-attention mechanism (SWHSA).

#### 3.3.1. Multi-Scale Self-Attention Enhanced Feed-Forward Networks (MS-FN)

**Figure 4:**
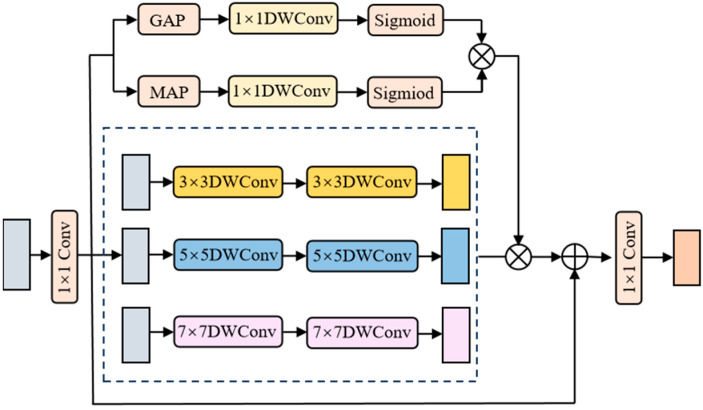
Architecture of the MS-FN.

As features propagate through the feed-forward unit, the hidden layer comprises numerous channels. Conventional feed-forward networks often struggle to aggregate this rich channel information effectively, leading to information loss over multiple layers. To address this issue, we design MS-FN, which employs deep convolutions with kernel sizes {3, 5, 7} to capture receptive field information at multiple scales from the input features. In parallel, SE channel attention assigns higher weights to salient channels and lower weights to redundant ones. Finally, a residual connection preserves features from preceding layers, mitigating vanishing gradients. Figure 4 depicts the MS-FN architecture.

#### 3.3.2. Shifted-Window Multi-Head Self-Attention (SWHSA)

When directly applying the original Swin Transformer shifted-window mechanism to time–frequency images of rotating machinery, the cross-window interaction may be insufficient for capturing elongated or directionally distributed fault patterns, and the fixed shifting strategy may be less adaptive to domain specific feature distributions under noise. To enhance cross-window information exchange while keeping local modeling capability, we propose an improved window shifting strategy that emphasizes transversal interaction, enabling more effective propagation of discriminative features across adjacent windows for fault-relevant structures.

**Figure 5:**
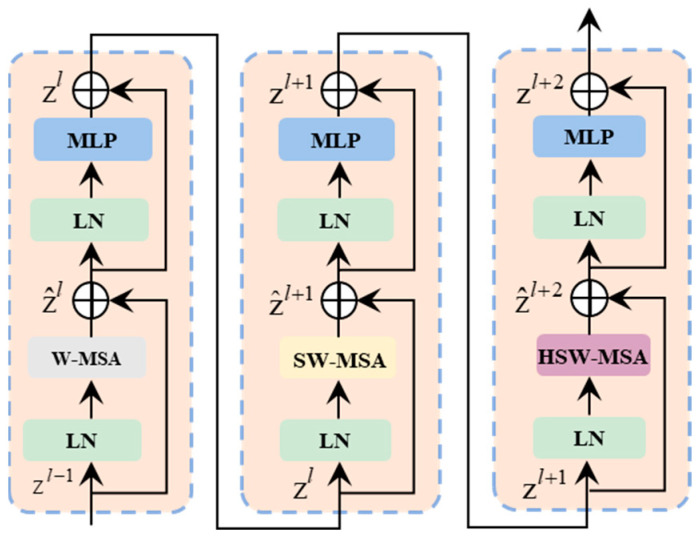
Architecture of three consecutive Transformer blocks.

As shown in Figure 5, the proposed method adopts architecture consisting of three consecutive Transformer modules stacked sequentially to achieve hierarchical feature extraction and optimization. Each Swin Transformer block is built around window-based MHSA and includes a multilayer perceptron (MLP) with layer normalization (LN) layers. Specifically, LN is applied around the MHSA and MLP submodules, and residual connections enable cross-layer feature propagation. This design improves training stability and feature representational capacity while mitigating vanishing gradients in deep networks. To enhance information exchange across windows, we propose a transversal shifted window multi-head self-attention (TSW-MHSA) module as an improvement over conventional MHSA. By optimizing the window shifting strategy, the module achieves efficient cross-window feature propagation and enhances the flexibility and adaptability of self-attention computations. In turn, this strengthens feature representational capacity and significantly improves task performance and prediction accuracy.

**Figure 6:**
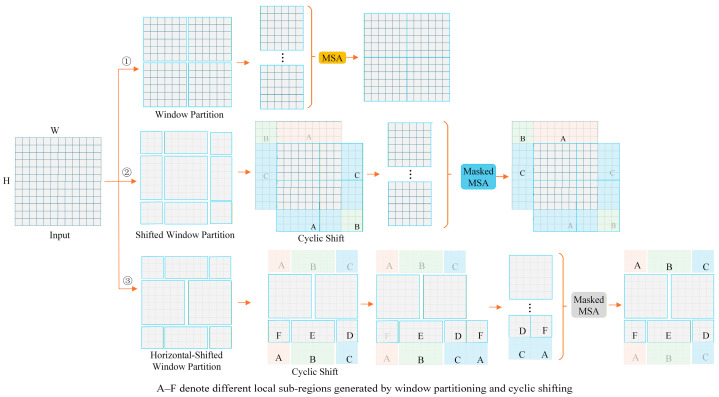
Architecture of variable Swin Transformer shifted window self-attention mechanism.

Following the Swin Transformer design, window shifting partitions each original window into multiple subwindows. This mechanism increases the number of windows stepwise, yielding a finer-grained network for feature extraction and interaction. Under lateral displacement, the increase in window count lies within the range $\frac{H}{N}\times \frac{W}{N}$ to $\frac{H}{N}\times (\frac{W}{N}+1)$. The increase in window count arises from partitioning and rearranging the original windows. Introducing a lateral window shifting mechanism expands the model’s effective receptive field, substantially improving its ability to represent and extract complex features. However, increasing the number of windows inevitably raises computational complexity, because each additional window incurs multiple MHSA computations. To balance model performance and computational cost, we apply dynamic shifting and rearrangement strategies to the sliding window, consolidating smaller windows into standard-sized windows. By performing self-attention on the restructured standard windows and precisely scheduling compute units, the total number of multi-head self-attention operations remains constant. This strategy eliminates redundant computations introduced by the sliding window mechanism, reduces the model computational load, and achieves a favorable trade-off between feature extraction capacity and computational efficiency. Figure 6 illustrates the operational pipeline.

The first panel of Figure 6 illustrates the baseline window partitioning scheme, which uses the top left pixel of the input feature map as the origin. At this stage, the 8 × 8 feature map is uniformly partitioned into four non-overlapping 4 × 4 windows. In the absence of cross-window information exchange, inter-feature correlations cannot be fully captured, limiting feature extraction under this segmentation. The second panel depicts the shifted window partitioning module, which employs a window configuration shifted relative to the preceding layer.

The split windows are shifted at the pixel level along the horizontal and vertical axes, creating partially overlapping regions between adjacent windows. This adjustment enhances the model’s ability to capture inter-window correlations via controlled overlap. The third panel depicts the horizontally shifted window partition module: each window is split horizontally, the upper half is shifted downward, and it is merged with the adjacent lower half subwindow, thereby reorganizing the window layout. The fourth panel shows a laterally shifted window segmentation module that again splits each window horizontally, shifts the upper half downward, and merges it with the adjacent lower half subwindow to further restructure the windows. Together, these operations endow the improved Swin Transformer with a dynamic window partitioning mechanism that significantly improves feature extraction efficiency.

Based on the aforementioned sliding window partitioning strategy, the computation flow of the improved Swin Transformer block is as follows:

(10) $$\begin{array}{l} {\hat{z}}^{l}=W-MSA(LN({\hat{z}}^{l-1}))+{\hat{z}}^{l-1}\\ z^{l}=MLP(LN({\hat{z}}^{l}))+{\hat{z}}^{l}\\ {\hat{z}}^{l+1}=SW-MSA(LN({\hat{z}}^{l}))+{\hat{z}}^{l}\\ z^{l+1}=MLP(LN({\hat{z}}^{l+1}))+{\hat{z}}^{l+1}\\ {\hat{z}}^{l+2}=TSW-MSA(LN({\hat{z}}^{l+2}))+{\hat{z}}^{l+2}\\ z^{l+2}=MLP(LN({\hat{z}}^{l+2}))+{\hat{z}}^{l+2} \end{array}$$

During computation, windowed multi-head self-attention (W-MSA), shifted window multi-head self-attention (SW-MSA), and TSW-MSA correspond, respectively, to conventional window partitioning, the baseline Swin Transformer shifted window partitioning, and transverse shift partitioning, thereby implementing a family of windowed MSA operators.

Standard Swin Transformer employs an MLP-based feed-forward unit and a fixed shifted-window scheme, which may not sufficiently aggregate multi-scale information across channels or capture cross-window dependencies in non-stationary fault patterns. In MSSwin-T, MS-FN introduces multi-scale convolutional modeling with dual attention to strengthen channel aggregation and expand the receptive field, while the improved shifted-window attention enhances cross-window interaction in a more task specific manner. These modifications make the backbone more suitable for rotating machinery diagnosis under noisy and small-sample conditions.

### 3.4. Overall Structure and Algorithmic Flow

**Figure 7:**
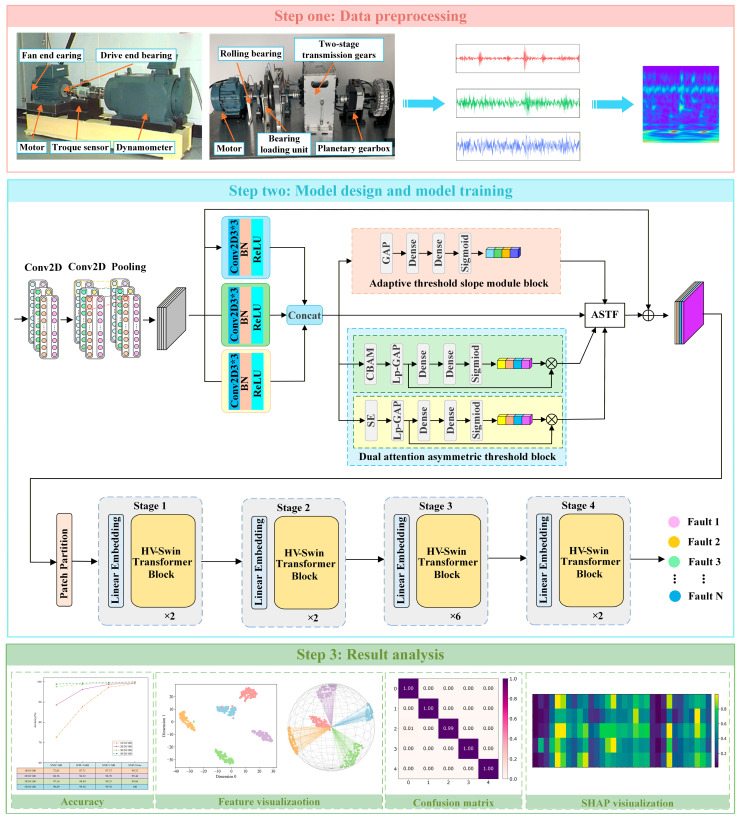
The proposed method fault diagnosis framework.

This paper proposes an intelligent rolling bearing fault diagnosis method based on explainable multi-source information fusion under small sample conditions. The overall structure is shown in Figure 7. In Figure 7, the SMIF-SRBFD process can be summarized into the following three steps: data preprocessing, model design and training, and result analysis.

Step 1: Data preprocessing. Vibration signal data is collected from the Case Western Reserve University (CWRU) and self-built PT890 datasets, divided into samples using a sliding window approach, and then converted into two-dimensional time–frequency signals via the MSDIFM.

Step 2: Model design and training. Input data processed by the multi-sensor data fusion module are fed into the network. Convolutional layers and MASRSB extract shallow features of fault information, while MSSwin-T learns deep features to enhance the network’s feature representation capability. Finally, the model achieving the highest accuracy on the validation set during iteration is selected as the training model.

Step 3: Result analysis. Input the test set into the trained model to obtain fault diagnosis results. Conduct multi-angle visualization analysis using t-SNE and confusion matrices. The results demonstrate that the model maintains excellent performance and robustness even in small samples and noisy environments.

The detailed algorithm flow of the method proposed in this paper is shown in Algorithm 1.
**Algorithm 1:** Training and testing process based on the proposed method**Input:** Multi-sensor time–frequency fusion images on training set $S_{train}={\left\{\left.x_{i},y_{i}\right\}\right.}_{i=1}^{n}$ and test set $S_{test}={\left\{\left.x_{j}\right\}\right.}_{j=1}^{m}$ **Output:** Fault diagnosis results on the test dataset ${\left\{\left.y_{j}\right\}\right.}_{j=1}^{m}$ **Initialization:** Initialize parameters of MSDIFM, MASRSB, MSSwin-T and classifier using random values. **Forward Propagation** 1. For epoch = 1 to epochs do 2. Generate fused time–frequency images via MSDIFM 3. Perform forward calculation on the time–frequency images input network 4. Calculate the cross-entropy loss function **End for** **Back Propagation** Compute the network gradient using Adam optimizer and update network parameter Until epoch = epochs Use trained neural networks for fault diagnosis

## 4. Experiments and Results Analysis

To validate the effectiveness and generalization capability of the proposed method, this paper employs the following two bearing datasets: the CWRU [28] bearing dataset and a self-created PT890 bearing dataset. 

### 4.1. CWRU Dataset Validation Results and Analysis

#### 4.1.1. Dataset Description

To evaluate the fault diagnosis performance of the proposed model, we use the CWRU bearing dataset. This dataset is the bearing dataset provided by CWRU in the United States. The test bearing is an SKF 6205, and the sampling frequency is 12 kHz. Faults are machined by electrical discharge machining with diameters of 0.007 inches, 0.014 inches, and 0.021 inches. Four bearing conditions are included: normal, ball fault, inner fault, and outer ring fault. Data are collected under four operating conditions: 0HP, 1HP, 2HP, and 3HP, by varying the load and speed. According to the location of the faults and the size of the faults, this paper chooses five fault states for each working condition to conduct experiments, and the specific faults and labels are shown in Table 1.

For each fault type, 200 multi-channel data fusion images are generated: 50 for training, 50 for validation, and 100 for testing. To evaluate fault diagnosis performance under limited sample conditions, four training subsets per class are randomly sampled with sizes of 10, 20, 30, and 50, respectively. Each subset is paired with an equal number of validation samples, while the test set remained fixed at 100 samples per fault type.

#### 4.1.2. Data Preprocessing

Assuming there are n sensors, with data represented as $g_{1},g_{2},\ldots ,g_{n}$, the main steps for processing the data are as follows:

Step 1: Data normalization. To prevent outliers in the dataset from affecting fault diagnosis, normalize the data from n sensors using the following mathematical expression.

(11) $$g^{\prime }=\frac{g_{i}-\min\left(g_{i}\right)}{\max\left(g_{i}\right)-\min\left(g_{i}\right)}$$

where $g^{\prime }$ represents the normalized data, $g_{i}$ represents the raw data, $\min\left(g_{i}\right)$ denotes the minimum value of $g_{i}$, and $\max\left(g_{i}\right)$ denotes the maximum value of $g_{i}$.

Step 2: Data fusion by combining data from n sensors into a matrix in parallel, yielding the data fusion matrix:

(12) $$N=\left[g_{1}^{\prime }|g_{2}^{\prime }|\ldots g_{n}^{\prime }|\right]$$

Step 3: Data segmentation by applying an overlapping sliding window sampling method to the data sampled by each sensor. The window size is 1024, and the stride is 64. This method effectively increases the number of samples.

Step 4: Perform dynamic weight calculation. Compute the variance of each sensor data segment as the basis for dynamic weights. First, calculate the variance of each sensor as $v_{1},v_{2}\ldots v_{n}$. To ensure the sum of weights equals 1, normalize the variance of each sensor. The normalized variance becomes the weight for each sensor, calculated as follows:

The total variance is $V=v_{1}+v_{2}+\ldots +v_{n}$, and the weights for each sensor are $w_{1}=\frac{v_{1}}{V},w_{2}=\frac{v_{2}}{V},\ldots ,w_{n}=\frac{v_{n}}{V}$.

Step 5: Generate the fused multi-sensor two-dimensional image. Perform CWT on the data from each sensor to convert the one-dimensional signals into two-dimensional images. Then, apply dynamic weighting based on the dynamic weights of each sensor to obtain the fused multi-sensor two-dimensional image.

**Figure 8:**
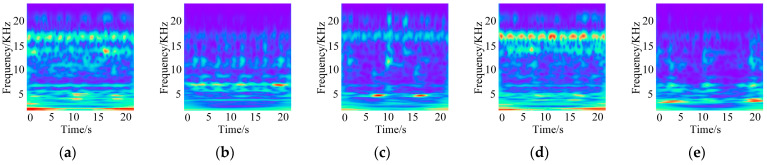
Fault diagnosis results: (**a**) ball-0.007; (**b**) inner-0.007; (**c**) inner-0.014; (**d**) outer-0.014; (**e**) outer-0.021.

In this experiment, acceleration data from three channels, namely the drive end accelerometer data (DE), fan end accelerometer data (FE), and base accelerometer data (BA), are fused to construct five two-dimensional time–frequency images corresponding to five typical faults, as shown in Figure 8. From left to right, the five images correspond to ball-0.007, inner-0.007, inner-0.014, outer-0.014, and outer-0.021, respectively. Different fault types and damage severities exhibit pronounced differences in the time–frequency energy distribution, resulting in well-separated feature patterns for subsequent fault identification.

#### 4.1.3. Results and Analysis of Fault Diagnosis Under Noisy and Small Sample Conditions

**Figure 9:**
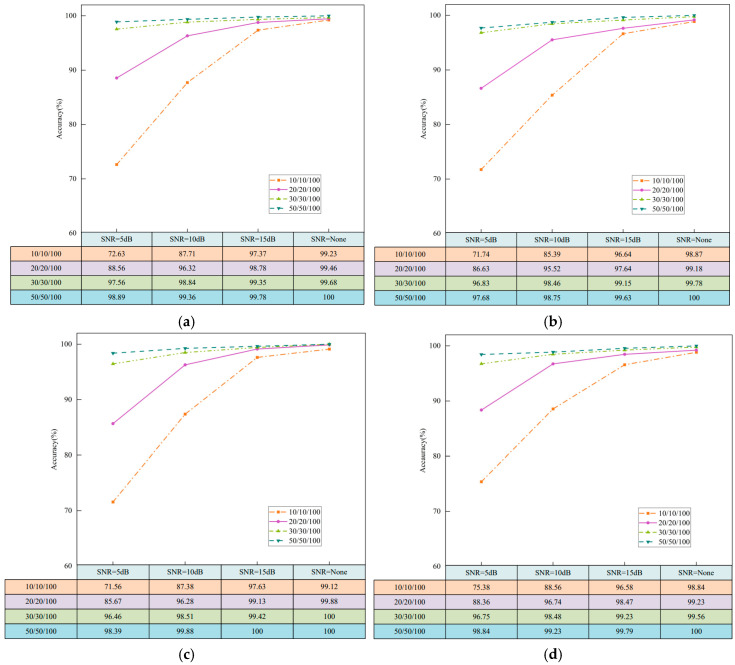
Fault diagnosis results: (**a**) 0HP; (**b**) 1HP; (**c**) 2HP; (**d**) 3HP.

In industrial settings, rotating machinery often operates in noisy environments in which interference obscures fault characteristics. Consequently, fault diagnosis under noisy conditions and limited sample regimes is challenging. To evaluate the method under small sample and noisy conditions, we use multi-sensor data fusion images across four operating conditions. Small sample settings include 10, 20, 30, and 50 samples per class. We add Gaussian white noise at Signal-to-Noise Ratios (SNRs) of 5, 10, and 15 dB, while an SNR label of none denotes no added noise. To ensure a fair comparison, each experiment is repeated five times, and the mean across runs is reported. Figure 9 reports results for all four operating conditions.

As shown in Figure 9, when only 10 samples per fault class are available, the diagnostic accuracy under the four operating conditions decreases to 72.63%, 71.74%, 71.56%, and 75.38%, respectively, after adding Gaussian white noise at 10 dB. This degradation is primarily due to the limited sample size, wherein noise obscures fault-related features in the original signal, leading the network to learn only a narrow set of features. Without added noise, the diagnostic accuracies under the four operating conditions are 99.23%, 98.87%, 99.12%, and 98.84%, respectively. This improvement stems from the proposed method’s intelligent multi-sensor data fusion, which enhances data quality, enabling the network to learn richer fault features and thereby improve diagnostic accuracy. As the sample size increases, diagnostic accuracy consistently improves across all settings. With 50 samples per fault class and an SNR of 10 dB, the average accuracy exceeds 98% under all four operating conditions; without added noise, accuracy reaches 100% across all cases. These results demonstrate strong noise robustness and excellent generalization of the proposed method.

**Figure 10:**
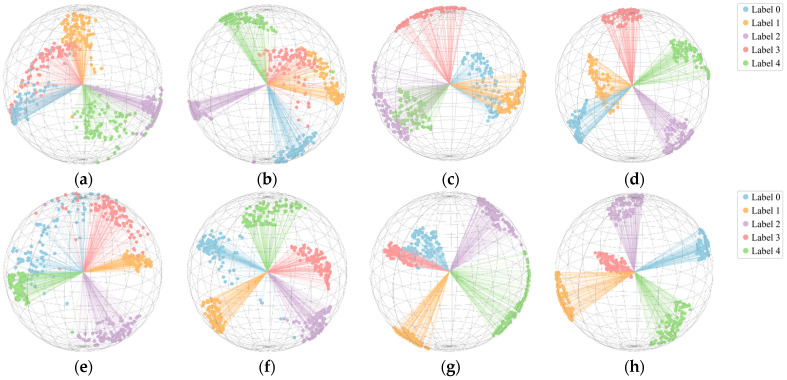
Visualization results of t-SNE: (**a**) SNR = 5 dB (1HP); (**b**) SNR = 10 dB (1HP); (**c**) SNR = 15 dB (1HP); (**d**) SNR = None (1HP); (**e**) SNR = 5 dB (2HP); (**f**) SNR = 10 dB (2HP); (**g**) SNR = 15 dB (2HP); (**h**) SNR = None (2HP).

To provide an intuitive assessment of diagnostic performance, we apply t-SNE at the classification layer to samples with 20 instances per class at load levels of 1 HP and 2 HP. The results are presented in Figure 10. At SNR = 5 dB, the t-SNE embedding shows weak cluster separation, and all five fault types exhibit varying degrees of misclassification. This suggests that noise obscures fault characteristics, leading to insufficient feature learning. As SNR increases, noise interference diminishes, facilitating feature extraction; for example, Figure 10b,c exhibit improved cluster compactness and separation. Without added noise, the proposed method exhibits strong fault classification capability: faults of the same type cluster tightly, whereas different types remain well separated, with no misclassification observed. The clustering results in Figure 10d,h are optimal, highlighting the method’s superior diagnostic performance under small sample conditions.

#### 4.1.4. Comparative Experimental Results and Analysis Under Small Sample Conditions

In practical settings, obtaining sufficient fault samples is often difficult. Therefore, it is critical that fault diagnosis methods maintain high accuracy under limited sample conditions. To evaluate the impact of small sample data on diagnostic accuracy, we compare them against the following baseline methods: MDCNN [29] converts vibration signals into two-dimensional images via the Markov Transition Field (MTF) and builds a fault diagnosis model based on a multi-dimensional attention mechanism, enabling fault classification under small sample conditions. SC-MSCNN [30] maps vibration signals to two-dimensional MTF images and constructs a multi-scale CNN for rolling bearing fault diagnosis under limited-sample scenarios. MTF-ResNet [31] maps vibration signals to two-dimensional images via the MTF and employs a deep ResNet for fault identification while preserving temporal dependencies. CovMNeT [32] uses a covariance metric network that enforces distributional consistency, improving small sample learning performance by preserving sample distribution characteristics. MLKFE [33] combines a Transformer-based model ensemble with Mahalanobis metric learning to exploit multi-scale features, enabling bearing fault diagnosis under small sample conditions. We vary the number of bearing fault samples per class to 10, 20, 30, and 50. Data at 2 HP operating conditions are used for testing. We compare our method against the five methods for performance evaluation. To more comprehensively analyze the performance of the proposed method, we used accuracy, precision, recall, and F1-score as evaluation metrics. Table 2 reports the experimental results.

As shown in Table 2, increasing the number of fault samples can enhance the performance of all methods. When the number of test set samples increases from 10 to 50, all methods show significant improvements in accuracy, precision, recall rate, and F1-score. We comprehensively evaluate the fault diagnosis performance of different methods under small sample conditions. Under all sample sizes, the proposed method consistently achieves the best results in the four evaluation indicators (accuracy, precision, recall rate, and F1-score), demonstrating excellent small sample learning ability and robustness. For each category, 10 samples are selected from the test set. The average fault diagnosis accuracy of the proposed method is 97.46%, the precision is 97.37%, the recall is 97.54%, and the F1-score is 97.45%. In contrast, the average fault diagnosis accuracy of MTF-ResNet, CovMNeT, MDCNN, MLKFE and SC-MSCNN is 80.58%, 70.86%, 76.52%, 85.53% and 78.83% respectively. With the increase in the number of samples, the diagnostic performance of all methods improves. Moreover, the proposed method maintains high accuracy, high recall rate and high F1-score while further improving its accuracy. When the number of test samples for each type of fault is 50, the performance of the proposed method in all four indicators (accuracy, precision, recall rate, and F1-score) is 100%. Compared with MTF-ResNet, CovMNeT, MDCNN, MLKFE, and SC-MSCNN, the proposed method achieves an average diagnostic accuracy that is higher by 4.74%, 11.62%, 10.07%, 3.65%, and 5.48%, respectively. These experimental results verify the advantages of the proposed method in small sample rolling bearing fault diagnosis. Overall, the proposed method maintains a high classification accuracy under different sample sizes, and performs stably in terms of precision, recall rate, and F1-score, demonstrating its good fault diagnosis effect and robustness under small sample conditions.

**Figure 11:**
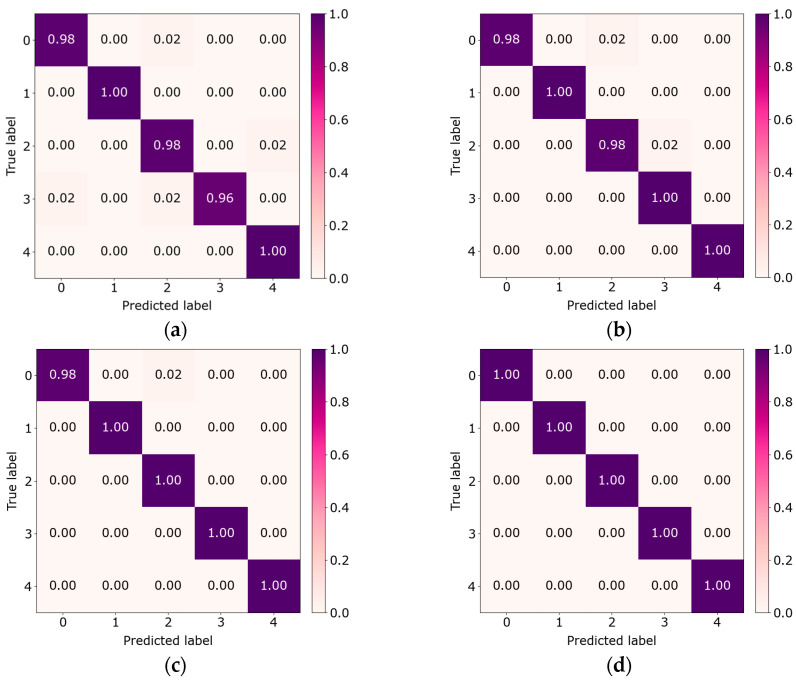
Confusion matrix results of different samples: (**a**) sample size: 10; (**b**) sample size: 20; (**c**) sample size: 30; (**d**) sample size: 50.

To provide an intuitive view of classification performance under varying sample sizes, we present confusion matrices of the proposed method’s predictions. Figure 11 presents the confusion matrices for the different sample sizes. With 10 samples per class, the model performs well, achieving 100% accuracy in classes 1 and 4. For classes 0, 2, and 3, some misclassifications remain, but overall accuracy is still high. With 50 samples per class, the proposed method reaches 100% accuracy, and no misclassifications are observed, indicating strong fault classification capability. These results indicate that accuracy improves markedly as sample size increases, approaching near-perfect performance even in small sample regimes. This reflects excellent generalization and stability in complex fault scenarios.

#### 4.1.5. The Influence of Different Time–Frequency Methods on the Results

**Figure 12:**
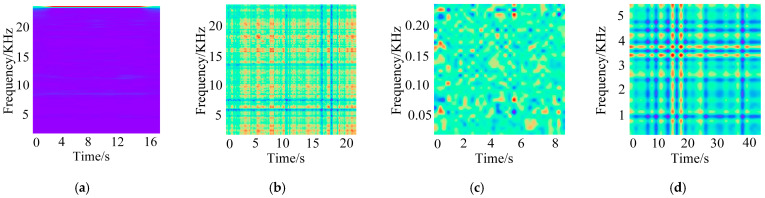
Time–frequency domain images of different generation methods: (**a**) STFT; (**b**) RP; (**c**) MTF; (**d**) GASF.

To assess how image generation choices affect fault diagnosis performance, this experiment uses 1 HP of ball-0.007 fault data to evaluate four techniques short time Fourier transform (STFT), recurrence plot (RP), MTF, and Gramian angle sum field (GASF). The representative feature maps generated by STFT, RP, MTF, and GASF are shown in Figure 12.

**Figure 13:**
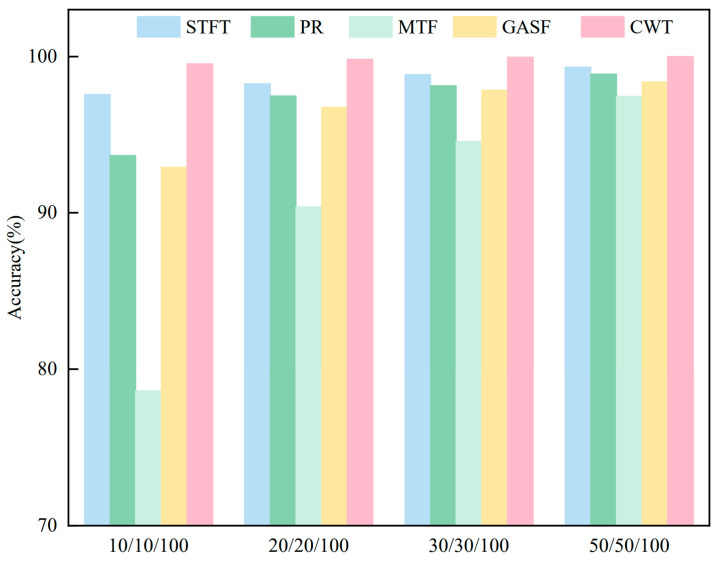
Fault diagnosis results for feature maps generated by different methods.

The multi-sensor signals are converted into multi-channel fused maps using the four image generation methods described above. These fused maps are then fed to the proposed method network for training and testing. The small sample partitions matched those used in the CWT experiments. Figure 13 reports the fault diagnosis results. As shown in Figure 13, with 10 samples per class, the average diagnostic accuracy for MTF maps is 78.63%, the lowest among the five methods. The average diagnostic accuracies for STFT, RP, GASF, and CWT are 97.56%, 93.67%, 92.93%, and 99.53%, respectively. These results indicate that converting multi-sensor signals into fused maps via the continuous wavelet transform better preserves time–frequency information. Consequently, the proposed network learns richer fault features, thereby enhancing diagnostic effectiveness.

#### 4.1.6. Analysis of Multi-Sensor Data Fusion Under Noisy Conditions

**Figure 14:**
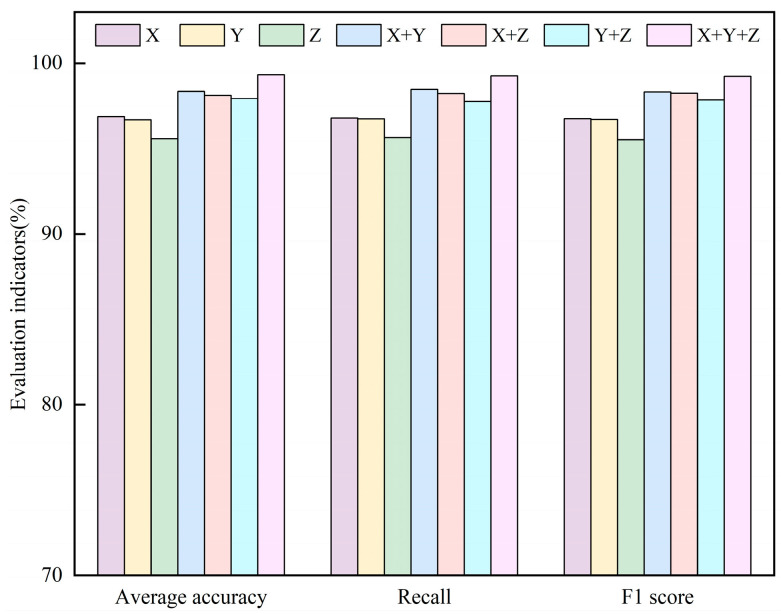
Multi-sensor data diagnosis results.

In industrial settings, rotating machinery operates in harsh environments where raw signals are contaminated by noise, and a single sensor often fails to capture sufficient information. To assess the effect of the intelligent multi-sensor data fusion module on multi-channel feature fusion under noise, this paper conducts 1-channel, 2-channel, and 3-channel input experiments. Here, DE represents the *X*-axis, FE represents the *Y*-axis, and BA represents the *Z*-axis. Figure 14 presents experimental design and results.

Experiments use 50 fault samples at 0 HP; Gaussian white noise at an SNR of 10 dB is added to simulate real-world conditions. In Figure 14, with single-sensor input, the diagnostic accuracies for *X*-axis, *Y*-axis, and *Z*-axis are 96.87%, 96.68%, and 95.56%, respectively. These results indicate that, under noise, single-sensor inputs to the proposed method struggle to identify all fault types, leading to some misclassifications. The BA shows the poorest performance, likely because its sensor is mounted at the bottom of the machine, where vibration amplitudes are lower. In contrast, the DE and FE sensors are mounted on opposite sides of the bearing housing, capturing more complementary fault information. With two-channel input, performance exceeds that of single-sensor input for any sensor pairing. The best two-channel result is obtained with the DE and FE sensors, yielding 98.35% accuracy. With three-sensor input, accuracy reaches 99.32%, with virtually no misclassifications. These results indicate that diagnostic accuracy increases as the number of input channels grows. This demonstrates that the proposed intelligent multi-sensor data fusion module effectively performs weighted fusion of complementary features while suppressing noise and irrelevant information.

#### 4.1.7. MSDIFM Weight Visualization and Explanation Analysis

**Figure 15:**
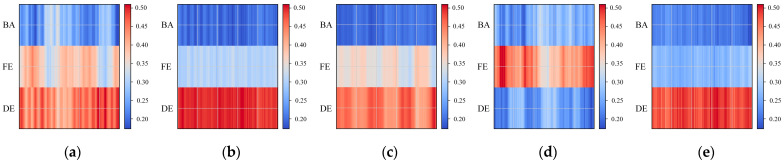
Weight visualization results: (**a**) ball; (**b**) inner-0.007; (**c**) inner-0.014; (**d**) outer-0.014; (**e**) outer-0.021.

To evaluate the adaptability and explanation of the proposed MSDIFM, fusion weight heatmaps are generated for five fault categories, as shown in Figure 15. The rows of the heatmap correspond to the three-channel sensors, while the columns represent the number of samples. Warmer colors indicate a greater weight for that channel in the fusion process. Weights are obtained through a dynamic weighting mechanism, where channel activity is characterized by segmented variance. After normalization, these weights are applied for pixel-level fusion. Overall, the weight distribution across different fault types exhibits significant variation. The color bands form a continuous strip along the horizontal axis rather than randomly scattered points, indicating stable weight estimation and the absence of single-channel collapse.

In terms of category performance, the ball-0.007 class exhibits a sustained increase in DE across multiple sample segments, indicating stronger rolling impact response at DE. Inner-0.007 and inner-0.014 both exhibit a significant dominance of DE, and as the fault size increases from 0.007 to 0.014 inch, the high weighting expands from discrete fragments to longer continuous bands, with BA and FE relatively suppressed. Outer-0.014 exhibits the strongest weighting band formed by FE, while DE and BA show significantly lower values, consistent with the empirical observation that outer faults exhibit stronger coupling with the frame. When the fault occurs at outer-0.021, the load shifts moderately from FE to DE, accompanied by a simultaneous increase across multiple channels. This indicates that the intense outer impact propagates along dual pathways: the shell and the shaft system.

The aforementioned patterns align with structural vibration mechanisms, demonstrating that MSDIFM can achieve adaptive fusion consistent with physical characteristics based on channel contribution. The heatmap exhibits stable color scales and temporal continuity, further validating the stability and robustness of weight estimation while providing a basis for subsequent sensor selection and deployment optimization. Experimental results also demonstrate that this module possesses significant adaptive adjustment capabilities. Under identical fault conditions, it intelligently weights the relative contributions of each signal source to state recognition, assigning differentiated weights to different channels. The weighting assigned to the same signal source can be dynamically adjusted across different health states based on its representational capability, enabling cross-fault adaptation in response to state changes. Overall, the MSDIFM demonstrates excellent effectiveness and explanation in bearing fault diagnosis.

#### 4.1.8. SHAP-Based Heatmap Explanation Analysis

**Figure 16:**
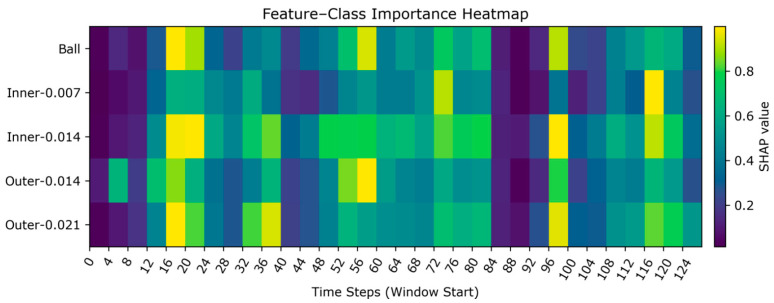
SHAP-based fault category-time step heatmap.

Figure 16 shows the distribution of the average absolute SHAP values for each fault category across different time steps. Brighter colors indicate a higher contribution of that time step to the classification of that category. Outer faults exhibit sparse yet sharp bright peaks, indicating that strong evidence can be obtained from a limited number of time positions. As the fault size increases from 0.014 to 0.021, both brightness and peak stability further enhance, consistent with the periodic impact mechanism triggered by the fixed load zone in the outer. Ball fault exhibits scattered hotspots of moderate intensity, requiring the model to accumulate evidence across multiple segments for identification. Inner faults appear smoother overall: inner-0.007 contributes weakly with high continuity, indicating more concealed features or shared characteristics with other classes. When dimensions increase to inner-0.014, more pronounced intermittent bright bands emerge, demonstrating that fault aggravation enhances separability.

Overall, the time-based SHAP attribution results indicate that the model selectively focuses on key temporal segments consistent with physical mechanisms. Outer faults manifest as sharp, punctate peaks; evidence of rolling faults appears dispersed; inner faults transition from weak, smooth patterns to intermittent intensification as dimensions increase. This diagram clarifies “when and why a fault is classified as a specific type,” providing a basis for subsequent fault diagnosis. Since the heatmap displays each fault category row by row, every row clearly indicates the time position with the highest contribution for that category. This dependency pattern aligns with the traditional impact mechanism of bearings, further validating the model’s mechanism of focusing on high contribution evidence during critical time segments. This enhances the model’s explanation.

#### 4.1.9. Explanation Analysis Using Occlusion-Based Spatiotemporal Heatmaps

**Figure 17:**
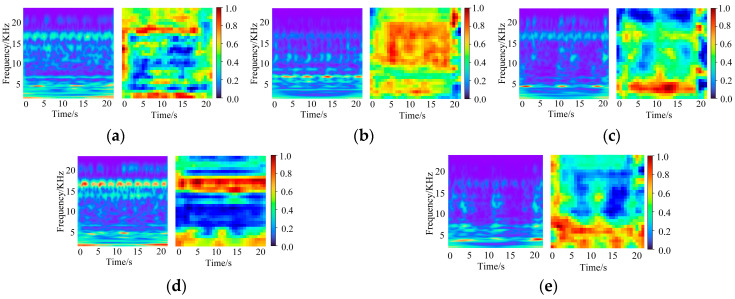
Occlusion-based spatiotemporal heatmap: (**a**) ball-0.007; (**b**) inner-0.007; (**c**) inner-0.014; (**d**) outer-0.014; (**e**) outer-0.021.

As shown in Figure 17, an occlusion-based time–frequency heatmap is presented to analyze the discrimination criteria for five faults: ball-0.007, inner-0.007, inner-0.014, outer-0.014, and outer-0.021 within the CWRU dataset. The occlusion heatmap is generated using the occlusion sensitivity method. A sliding window with a size of 8 and a stride of 4 is applied to the time–frequency map of the network input, progressively zeroing out local regions. The reduction in the predicted probability of the target fault category relative to the complete sample is recorded. By calculating the cumulative average of probability reductions across all window positions and normalizing the results to the (0, 1) interval, we obtain an importance score for each pixel. The higher the score value, the more significant the confidence in predicting the target fault category is weakened when that region is obscured, making it more critical for the classification of that category.

As shown in Figure 17, the distribution of sensitive regions in the time–frequency domain varies significantly across different fault types and fault sizes. For the ball-0.007, the heatmap exhibits relatively scattered bright spots in the mid-frequency and mid-to-late time domains, indicating that the model must integrate multiple dispersed impact feature fragments to complete rolling fault identification. At inner-0.007, the significant regions are primarily concentrated within block-like areas in the mid-frequency band, exhibiting a relatively compact spatial distribution. This indicates that the model’s discriminative evidence for this type of fault dependency is more concentrated. As the inner fault size increases to inner-0.014, both the amplitude and coverage of the highlighted region significantly improve, forming a more continuous hotspot band at low to mid frequencies. This indicates that stronger periodic impact energy is being used by the network as the primary discrimination criterion.

In contrast, the occlusion time–frequency heatmap of outer-0.014 exhibits a more typical horizontal band structure, with bright regions continuously distributed along specific frequency bands while other bands show weaker responses. This indicates that the detection of outer faults primarily relies on the characteristics of a relatively stable resonant frequency band. As the fault further deteriorates to outer-0.021, the high sensitivity region expands significantly in both the time and frequency domains. A more prominent bright block appears at the boundary between low and mid frequencies, indicating that severe outer faults exert a substantial influence on vibration responses across a broader time–frequency range. The model’s decision-making pattern evolves from relying on local features to comprehensively characterizing and utilizing global impact patterns.

Overall, the results from the occlusion-based time–frequency heatmap demonstrate that the proposed model effectively focuses on impact energy concentration features closely related to the failure mechanism when making diagnostic decisions, rather than random noise or regions lacking physical significance. Simultaneously, the distinct patterns of sensitive regions corresponding to different fault types and sizes demonstrate excellent distinguishability, thereby validating the physical plausibility of the constructed fault diagnosis model and the reliability of its diagnostic results from an explanation perspective.

#### 4.1.10. Results of Ablation Experiments

To verify the contributions of each module and their impact on the fault diagnosis results, several different network structures are established in this experiment: method 1 represents using single sensor data to generate images to replace the intelligent fusion of multi-sensor data images; other network structures remain unchanged. Method 2 indicates that, while keeping the network structure unchanged, the traditional residual contraction block is replaced by the MASRSB; other network structures remain unchanged. Method 3 represents using the basic Swin Transformer to replace the multi-scale Swin Transformer; other network structures remain unchanged. In this experiment, the experimental data under 0HP are selected. When the number of training sets for each fault is 20, the experimental results are shown in Table 3.

As shown in Table 3, the feature images generated from single-sensor data achieve a fault diagnosis accuracy of 98.35% under noise-free conditions, indicating satisfactory diagnostic capability. However, the performance degrades markedly after noise is introduced: at SNR = 5 dB, the diagnosis accuracy drops to 73.82%, whereas the proposed method still reaches 88.56%. These results suggest that the time–frequency images obtained via intelligent multi-sensor data fusion can more comprehensively characterize fault-related vibration signatures, enabling the network to learn richer fault features even under severe noise interference. At SNR = 10, 15 and 20 dB, method 2 achieves fault diagnosis accuracies of 82.56%, 93.28% and 97.63%, respectively, whereas the proposed method attains average accuracies of 88.56%, 96.32% and 98.78%. These results demonstrate that the MASRSB provides stronger noise immunity. At an SNR of 5 dB, the average fault diagnosis accuracy of method 3 drops to 68.73%, and its diagnostic performance under the other noise conditions is also inferior to that of the proposed method. These results indicate that the proposed multi-scale Swin Transformer improves fault diagnosis performance by learning deep, discriminative fault features.

### 4.2. Self-Built PT890 Dataset

#### 4.2.1. Dataset Description

**Figure 18:**
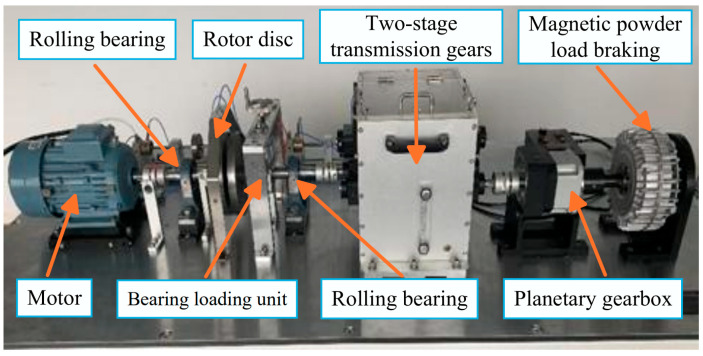
Experimental bench for the PT890 dataset.

To further validate the fault diagnosis performance of the proposed model, it is verified using a dataset from a self-built PT890 bearing fault experimental platform. This equipment is manufactured by Valenian Company in Suzhou, China. As shown in Figure 18, the experimental platform consists of a 2.2 kW motor, bearing unit, loading device, and other components. The bearing model under test is UPH206. Data are collected at a sampling frequency of 10 kHz. Sensors capture data along the X, Y, and Z axes. Fault dimensions include 0.09 inch, 0.14 inch, 0.25 inch, and 0.28 inch. Five bearing conditions are identified: normal condition, ball fault, inner fault, outer fault, and compound fault. Five positions are simulated under normal conditions, and acquire signals at rotational speeds of 1825 r/min, 1600 r/min, 1375 r/min, and 1150 r/min.

According to the location of the faults and the size of the faults, this paper chooses five fault states for each working condition to conduct experiments, and the specific faults and labels are shown in Table 4. All other settings match those of the CWRU dataset.

#### 4.2.2. Results of Small Sample Fault Diagnosis

To evaluate generalization under small sample scenarios, the experiment selected data at rotational speeds of 1825 r/min, 1600 r/min, 1375 r/min, and 1150 r/min for testing. The results are shown in Table 5.

As shown in Table 5, the overall accuracy of the proposed method decreases slightly when the laboratory-generated dataset is incorporated. With 10 samples per class, the average accuracies across the four rotational speeds are 97.63%, 97.78%, 97.46%, and 97.84%, respectively. With 50 samples per class, the average accuracies across the four rotational speeds reach 99.84%, 99.87%, 99.78%, and 99.89%. These results indicate that the method maintains strong generalization even under small sample conditions.

#### 4.2.3. Comparative Method Analysis Under Small Sample Conditions

To further verify the superiority of the proposed method under the condition of small sample size, a comparative experiment is conducted at 1600 r/min, and it is compared with five advanced methods. Diagnostic performance is comprehensively evaluated using four indicators: accuracy, precision, recall, and F1-score. The experimental results are shown in Table 6.

Overall, as the sample size increases, all the methods show an upward trend in the four indicators. When the test sample size for each type of fault is 10, the accuracy rate, accuracy rate, recall rate and F1-score of the proposed method reach 97.78%, 97.67%, 97.85% and 97.76% respectively. These four indicators are significantly better than those of the comparison methods, demonstrating their excellent diagnostic performance and stable discrimination ability under small sample conditions. In contrast, the accuracy rates of MTF-ResNet, CovMNeT, MDCNN, MLKFE, and SC-MSCNN are 79.47%, 67.75%, 73.67%, 90.63%, and 77.63% respectively. With the increase in the number of fault samples, the diagnostic performance of all methods shows a continuous improvement trend. When the test sample size for each type of fault is 50, the accuracy of the proposed method further increases to 99.87%, and the precision, recall, and F1-score reach 99.84%, 99.89%, and 99.86% respectively, indicating a more balanced and stable classification performance. In conclusion, the above comparison results fully demonstrate the superiority of the proposed method in the small sample rolling bearing fault diagnosis task and its good robustness and generalization ability. When the test sample size for each type of fault is 10, the accuracy rate, accuracy rate, recall rate and F1-score of the proposed method reach 97.78%, 97.67%, 97.85% and 97.76% respectively.

**Figure 19:**
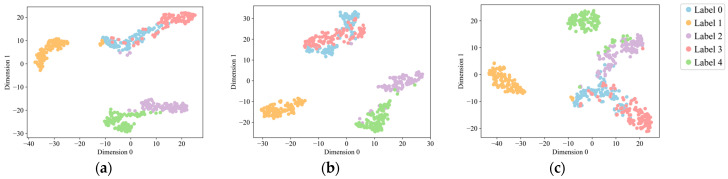

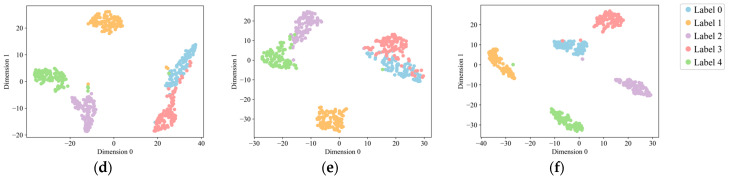
t-SNE visualization for different methods: (**a**) MTF-ResNet; (**b**) CovMNeT; (**c**) MDCNN; (**d**) MLKFE; (**e**) SC-MSCNN; (**f**) proposed.

In order to visualize the feature extraction capability of different methods, 1600 r/min and 20/20/100 are selected for t-SNE visualization. Figure 19 presents the t-SNE results for the different methods. The visualizations show that CovMNeT and MDCNN exhibit inter-class overlap and blurred decision boundaries among fault types in feature space. This indicates that, under small sample conditions, both models have limited feature representation capacity, leading to biased predictions. SC-MSCNN and MTF-ResNet achieve effective separation for most class boundaries in feature space; however, classes 0 and 3 still exhibit substantial overlap, making them difficult to distinguish reliably. MLKFE effectively separates most classes, but a small cluster overlap remains between classes 0 and 3, likely stemming from similar fault characteristics and resulting in insufficient inter-class discrimination. The proposed method clearly separates all five classes in feature space: same class samples cluster densely, different classes are well separated, and no misclassifications are observed. These visualizations demonstrate strong feature extraction and discrimination capability. The proposed method distinguishes different fault types while maintaining consistent, accurate recognition for similar faults. These findings further validate the method’s reliability and performance advantages under small sample conditions.

#### 4.2.4. Confusion Matrix Results Analysis

**Figure 20:**
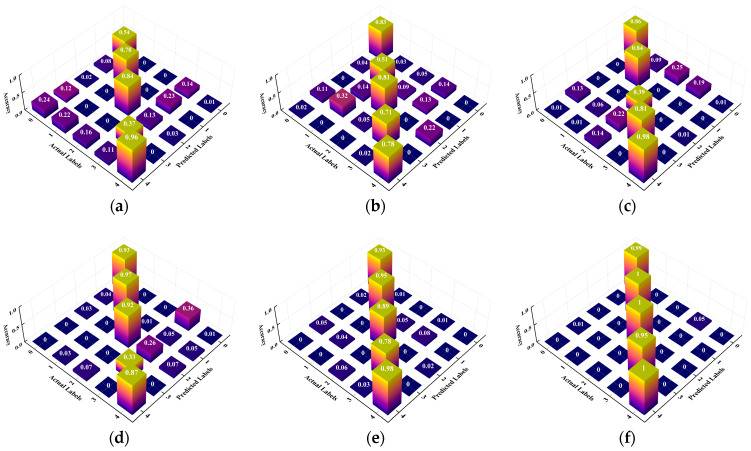
Confusion matrix for different methods: (**a**) MTF-ResNet; (**b**) CovMNeT; (**c**) MDCNN; (**d**) MLKFE; (**e**) SC-MSCNN; (**f**) proposed.

To more intuitively demonstrate the fault diagnosis performance of different methods, a confusion matrix analysis is conducted under 10/10/100 conditions. Figure 20 presents the visualization results of the confusion matrices for various methods. In Figure 20, with only 10 samples per class, the misclassification rates of MTF-ResNet, CovMNeT, MDCNN, and SC-MSCNN increase markedly, with overall diagnostic accuracies of 79.57%, 67.75%, 73.67%, and 77.63%, respectively. These results indicate that, under small sample conditions, the discriminative capacity of these methods is limited. MLKFE attains 90.63% accuracy, indicating strong performance even under small sample conditions. The proposed method achieves 98.76% accuracy, significantly higher than the baselines. Overall, the proposed method attains high diagnostic accuracy under small sample conditions and demonstrates excellent generalization.

#### 4.2.5. SHAP-Based Heatmap Explanation Analysis

**Figure 21:**
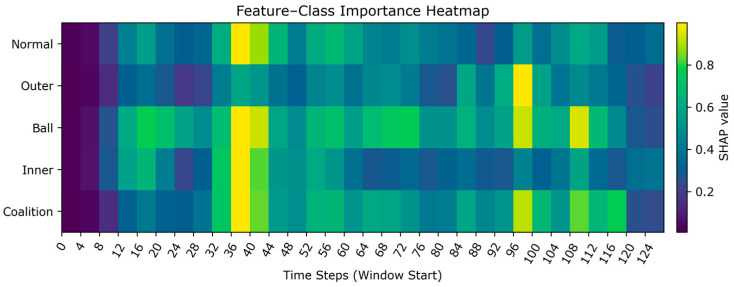
SHAP-based fault category-time step heatmap.

Figure 21 presents a SHAP-based fault category-time step heatmap, in which the distribution of discriminative evidence for each fault class varies substantially over time. Brighter colors indicate higher mean absolute SHAP values for that class at the corresponding time step. The outer fault exhibits sparse but sharp peaks near time steps 80 and 108, indicating that a small number of time positions provide strong discriminative evidence. Ball fault shows relatively dispersed hotspots, requiring the model to aggregate multiple moderately intense segments to achieve reliable recognition. Inner fault displays relatively weak and smooth responses overall, suggesting that their characteristics are more concealed or partially shared with other classes. Compound fault exhibits broadband, continuous brightening, reflecting the accumulation of multi-source impacts that provide discriminative evidence over a longer time span. Normal samples remain predominantly low, with only localized, slight elevations consistent with a normal state lacking prominent impact characteristics.

Overall, the SHAP-based fault category time step heatmap shows that the model selectively focuses on high contribution evidence during critical time segments consistent with physical mechanisms: outer fault exhibits points like peaks, ball fault shows dispersed evidence, and compound fault manifests as broad, sustained highlights. This time-based attribution not only identifies the temporal segments and their relative contributions that drive the model’s decision but also increases temporal resolution at high contribution time steps. It employs a weighting mechanism to emphasize critical segments while applying targeted enhancement in low SNR regions. As a result, the model’s decision process becomes more transparent and explainable, enhancing its explainability without sacrificing performance.

## 5. Conclusions

An explainable intelligent fault diagnosis for rotating machinery via multi-source information fusion under noisy environments and small sample conditions is proposed. In the proposed method, a MSDIFM is designed, employing dynamic weighting for pixel-level fusion of multi-sensor CWT time–frequency maps. This approach adaptively enhances high information channels while improving feature separability and recognition robustness. Second, a MASRSB is constructed to separate positive and negative features and apply dynamic threshold adjustment, thereby effectively enhancing feature representation capability and discrimination performance. Subsequently, the MSSwin-T backbone architecture is designed. Within this framework, the MS-FN module employs deep convolutions with varying kernel sizes combined with a dual-channel attention mechanism to highlight key information and suppress redundant features, thereby enhancing forward representation capabilities. Concurrently, the SWHSA module strengthens cross-window interactions through directional window shifting and window reorganization, further elevating the model’s overall expressive power. Finally, experimental validation is conducted on the CWRU dataset and the self-built PT890 dataset. Results indicate that under small sample conditions, the proposed method achieves an average diagnostic accuracy of 99.26%, representing a 6.83% improvement over existing methods. Even under small sample conditions with noise interference, the proposed method maintains an average diagnostic accuracy of 94.58%. These results demonstrate that the proposed method not only effectively extracts key fault features but also exhibits both excellent diagnostic performance and robustness.

Although the proposed method has achieved good diagnostic results, it also increases the complexity of the model while enhancing the diagnostic performance through the MASRSB and MSSwin-T modules. Therefore, in future research, we will focus on the lightweighting study of the model.

## Figures and Tables

**Table 1:** Division of CWRU.

Load (HP)	Fault Location	Fault Diameter	Label	Train/Valid/Test Samples
1790 (0HP)	Ball	0.007	0	10/10/100
1772 (1HP)	Inner	0.007	1	20/20/100
1750 (2HP)	Inner	0.014	2	30/30/100
1730 (3HP)	Outer Outer	0.014 0.021	3 4	50/50/100

**Table 2:** Comparison of fault diagnosis accuracy among different methods at various sample sizes.

Model	Indicators	10/10/100	20/20/100	30/30/100	50/50/100
MTF-ResNet	Accuracy	80.58 ± 0.56	87.82 ± 0.48	91.37 ± 0.38	95.26 ± 0.26
	Precision	80.36 ± 0.38	87.86 ± 0.58	91.48 ± 0.23	95.35 ± 0.24
	Recall	80.23 ± 0.52	87.75 ± 0.34	91.34 ± 0.34	94.28 ± 0.32
	F1-score	80.29 ± 0.46	87.80 ± 0.42	91.41 ± 0.28	94.81 ± 0.27
CovMNeT	Accuracy	70.86 ± 1.23	78.73 ± 0.85	85.36 ± 0.58	88.38 ± 0.46
	Precision	70.48 ± 1.15	77.85 ± 0.91	85.24 ± 0.63	87.45 ± 0.41
	Recall	69.86 ± 1.27	78.52 ± 0.83	85.63 ± 0.56	88.36 ± 0.47
	F1-score	70.17 ± 1.21	78.18 ± 0.86	85.43 ± 0.60	87.90 ± 0.43
MDCNN	Accuracy	76.52 ± 0.86	82.83 ± 0.64	88.83 ± 0.47	89.93 ± 0.38
	Precision	76.36 ± 0.76	82.67 ± 0.58	88.75 ± 0.53	90.14 ± 0.29
	Recall	77.13 ± 0.91	83.13 ± 0.47	88.86 ± 0.48	89.87 ± 0.36
	F1-score	76.74 ± 0.83	82.90 ± 0.52	88.80 ± 0.51	90.00 ± 0.32
MLKFE	Accuracy	85.53 ± 0.68	91.34 ± 0.43	95.36 ± 0.34	96.35 ± 0.23
	Precision	84.96 ± 0.63	91.56 ± 0.35	95.54 ± 0.28	96.68 ± 0.19
	Recall	85.57 ± 0.55	90.84 ± 0.46	95.38 ± 0.37	96.23 ± 0.26
	F1-score	85.26 ± 0.58	90.20 ± 0.41	95.51 ± 0.32	96.45 ± 0.23
SC-MSCNN	Accuracy	78.83 ± 0.82	85.43 ± 0.67	89.83 ± 0.42	94.52 ± 0.35
	Precision	78.76 ± 0.86	85.35 ± 0.71	89.64 ± 0.39	94.45 ± 0.38
	Recall	78.92 ± 0.79	85.67 ± 0.63	89.79 ± 0.34	94.56 ± 0.31
	F1-score	78.84 ± 0.83	85.51 ± 0.66	89.71 ± 0.37	94.50 ± 0.34
Proposed	Accuracy	97.46 ± 0.21	99.36 ± 0.14	99.63 ± 0.08	100 ± 0.0
	Precision	97.37 ± 0.23	99.18 ± 0.15	99.67 ± 0.07	100 ± 0.0
	Recall	97.54 ± 0.18	99.25 ± 0.11	99.54 ± 0.10	100 ± 0.0
	F1-score	97.45 ± 0.21	99.21 ± 0.13	99.60 ± 0.09	100 ± 0.0

**Table 3:** Fault diagnosis accuracy of different methods.

Methods	Accuracy/%			
	SNR = 5 dB	SNR = 10 dB	SNR = 15 dB	SNR = None
Method 1	73.82	89.54	96.73	98.35
Method 2	82.56	93.28	97.63	98.74
Method 3	68.73	87.86	94.68	97.53
Proposed	88.56	96.32	98.78	99.46

**Table 4:** Division of the PT890 dataset.

Working Condition	Fault Location	Fault Diameter	Label	Train/Valid/Test Samples
1825	Normal	/	0	10/10/100
1600	Ball	0.14	1	20/10/100
1375	Inner	0.09	2	30/30/100
1150	Outer	0.25	3	50/50/100
	Compound	In0.14 and Ou 0.28	4	

**Table 5:** Fault diagnosis results.

Working Condition	Accuracy (%)			
	10/10/100	20/20/100	30/30/100	50/50/100
1850	97.63	98.45	99.48	99.84
1600	97.78	98.53	99.46	99.87
1375	97.46	98.38	99.39	99.78
1150	97.84	98.62	99.42	99.89

**Table 6:** Comparison of fault diagnosis performance of different methods at various sample sizes.

Model	Indicators	10/10/100	20/20/100	30/30/100	50/50/100
MTF-ResNet	Accuracy	79.47 ± 0.56	86.64 ± 0.45	92.84 ± 0.34	95.27 ± 0.25
	Precision	79.32 ± 0.38	86.67 ± 0.54	92.81 ± 0.32	95.23 ± 0.31
	Recall	79.39 ± 0.52	86.59 ± 0.38	92.91 ± 0.27	95.34 ± 0.23
	F1-score	79.35 ± 0.56	86.63 ± 0.45	92.86 ± 0.29	95.28 ± 0.26
CovMNeT	Accuracy	67.75 ± 1.19	77.35 ± 0.83	83.57 ± 0.47	85.63 ± 0.43
	Precision	67.46 ± 1.23	77.64 ± 0.76	83.78 ± 0.56	85.87 ± 0.37
	Recall	66.89 ± 1.14	76.83 ± 0.87	83.43 ± 0.44	85.56 ± 0.46
	F1-score	67.17 ± 1.16	77.22 ± 0.81	83.61 ± 0.49	85.67 ± 0.41
MDCNN	Accuracy	73.67 ± 0.82	81.43 ± 0.59	86.78 ± 0.54	88.63 ± 0.44
	Precision	74.23 ± 0.85	81.48 ± 0.62	86.83 ± 0.47	88.76 ± 0.41
	Recall	73.48 ± 0.74	81.67 ± 0.53	86.71 ± 0.58	88.58 ± 0.48
	F1-score	73.85 ± 0.79	81.59 ± 0.57	86.76 ± 0.52	88.67 ± 0.45
MLKFE	Accuracy	90.63 ± 0.38	92.72 ± 0.35	95.83 ± 0.32	97.35 ± 0.21
	Precision	90.45 ± 0.32	92.83 ± 0.31	95.78 ± 0.38	97.21 ± 0.18
	Recall	90.71 ± 0.41	92.65 ± 0.39	95.91 ± 0.29	97.43 ± 0.26
	F1-score	90.61 ± 0.35	92.74 ± 0.36	95.85 ± 0.33	97.32 ± 0.23
SC-MSCNN	Accuracy	77.63 ± 0.81	85.76 ± 0.65	89.82 ± 0.41	93.56 ± 0.36
	Precision	77.71 ± 0.86	85.65 ± 0.62	89.77 ± 0.36	93.47 ± 0.32
	Recall	77.52 ± 0.78	85.83 ± 0.71	89.85 ± 0.39	93.61 ± 0.39
	F1-score	77.61 ± 0.83	85.74 ± 0.66	89.81 ± 0.38	93.54 ± 0.35
Proposed	Accuracy	97.78 ± 0.22	98.53 ± 0.15	99.46 ± 0.07	99.87 ± 0.08
	Precision	97.67 ± 0.18	98.46 ± 0.17	99.38 ± 0.10	99.84 ± 0.06
	Recall	97.85 ± 0.23	98.51 ± 0.11	99.51 ± 0.06	99.89 ± 0.09
	F1-score	97.76 ± 0.20	98.49 ± 0.14	99.44 ± 0.08	99.86 ± 0.07

## Data Availability

Due to privacy restrictions, the data provided in this study are available on request from the first author.
